# Development of a radionuclide-labeled monoclonal anti-CD55 antibody with theranostic potential in pleural metastatic lung cancer

**DOI:** 10.1038/s41598-018-27355-8

**Published:** 2018-06-12

**Authors:** So Hee Dho, Soo Yong Kim, Chaeuk Chung, Eun Ha Cho, So-Young Lee, Ji Young Kim, Lark Kyun Kim, Sung-Won Min, Jichul Lee, Sung Hee Jung, Jae Cheong Lim

**Affiliations:** 1 Radioisotope Research Division, Department of Research Reactor Utilization, Korea Atomic Energy Research Institute, Daejeon, 34057 Republic of Korea; 2 Division of Pulmonary and Critical Care Medicine, Department of Internal Medicine, Chungnam National University Hospital, Daejeon, 35015 Republic of Korea; 3 Hormone Research Center, School of Biological Sciences and Technology, Chonnam National University, Gwangju, 61186 Republic of Korea; 4 Severance Biomedical Science Institute and BK21 PLUS project to Medical Sciences, Gangnam Severance Hospital, Yonsei University College of Medicine, Seoul, 06230 Republic of Korea; 5 SG Medical, Inc, Seoul, 05548 Republic of Korea

## Abstract

Decay-accelerating factor (CD55 or DAF) inhibits complement-dependent cytotoxicity. We determined that CD55 is overexpressed in 76.47% of human non-small cell lung cancer tissue specimens. We therefore developed a lutetium-177-labeled chimeric monoclonal antibody against CD55. CD55-specific single-chain variable fragment (scFv) was selected from a naïve chicken scFv phage-display library, converted to IgG, and radiolabeled with lutetium-177 to generate a ^177^Lu-anti-CD55 antibody. We then charaterized the biodistribution of this antibody in a mouse model of pleural metastatic lung cancer. The ^177^Lu-anti-CD55 antibody was primarily retained in tumor tissue rather than normal tissue. Treatment of the mice with ^177^Lu-anti-CD55 reduced the growth of lung tumors and improved median survival *in vivo* by two-fold compared to controls. Finally, ^177^Lu-anti-CD55 also enhanced the antitumor activity of cisplatin both *in vitro* and *in vivo*. These data suggest ^177^Lu-anti-CD55 antibody is a promising theranostic agent for pleural metastatic lung cancer.

## Introduction

Pleural metastasis is a frequent cause of pain and dyspnea in patients with advanced cancer. It is correlated with a worse prognosis in various tumors including lymphomas and carcinomas of the lung, breast, gastrointestinal tract, and ovaries^1,2^. Lung cancer, especially non-small cell lung cancer (NSCLC) is the most common (37.5%) cause of pleural metastasis including malignant pleural effusion^3^. Treatment of pleural metastasis consists of tube thoracostomy and pleurodesis using sclerosing agents such as talc and Viscum album^4^. However, in many cases, pleural metastases are refractory to these treatments because they are not primary systemic therapies that target metastatic lung cancer cells. More systemic intrathoracal chemotherapy including hyperthermic chemotherapy with cisplatin, doxorubicin, or mitomycin^5^ has been lagging behind because the result was controversial. To the best of our knowledge, there is no successful clinical study associated with systemic targeted immunotherapies on pleural metastasis. Targeted immunotherapies such as pembrolizumab have demonstrated efficacy in advanced lung cancer^6^. However, the efficacy is limited by poor penetration in solid tumors^7,8^. With the exception of hematological malignancies, immunotherapies must penetrate tissue to access target cells^9^. Catumaxomab is approved in the European Union for the treatment of malignant ascites^10^. The efficacy of catumaxomab is partly due to the efficient delivery of the drug to free circulating tumor cells in ascites fluid. Malignant ascites and malignant pleural effusions both contain floating tumor cells, which could be effectively targeted by immunotherapies.

The efficacy of cancer immunotherapy is enhanced by antibody-drug conjugates such as radionuclides. Zevalin (^90^Y-ibritumomab tiuxetan), a radioimmunotherapeutic, is widely used for the treatment of non-Hodgkin’s lymphoma^11^. Since ^177^Lu emits both γ- and β- radiation, which may be useful for imaging and treatment, respectively, ^177^Lu-labeled radioimmunotherapies have been investigated for theranostic applications^11,12^.

CD55 is a glycosylphosphatidylinositol-anchored protein that inhibits complement-mediated lysis through dissociation of the C3 and C5 convertases^13,14^. Inhibition of CD55 was shown to induce apoptosis or growth arrest as well as complement-dependent cytotoxicity^15^. CD55 is frequently overexpressed in lung cancer^16^, colorectal cancer^17–19^, gastric cancer^20^, breast cancer^21^, ovarian cancer^22^, leukemia^23^, and cervical cancer^24^. Because inhibition of CD55 promotes apoptosis, an anti-CD55 antibody has been administered in combination with rituximab, herceptin, or surgery, and as a monotherapy in lymphoma^25^ and gastric cancer^26^. However, the effects of CD55 immunotherapy or radioimmunotherapy have not been investigated in pleural metastatic lung cancer. Here, we developed and characterized a radionuclide-labeled anti-CD55 monoclonal antibody and evaluated it as a theranostic agent in pleural metastatic lung cancer.

## Results

### CD55 expression in human lung cancer tissue

**Figure 1:**
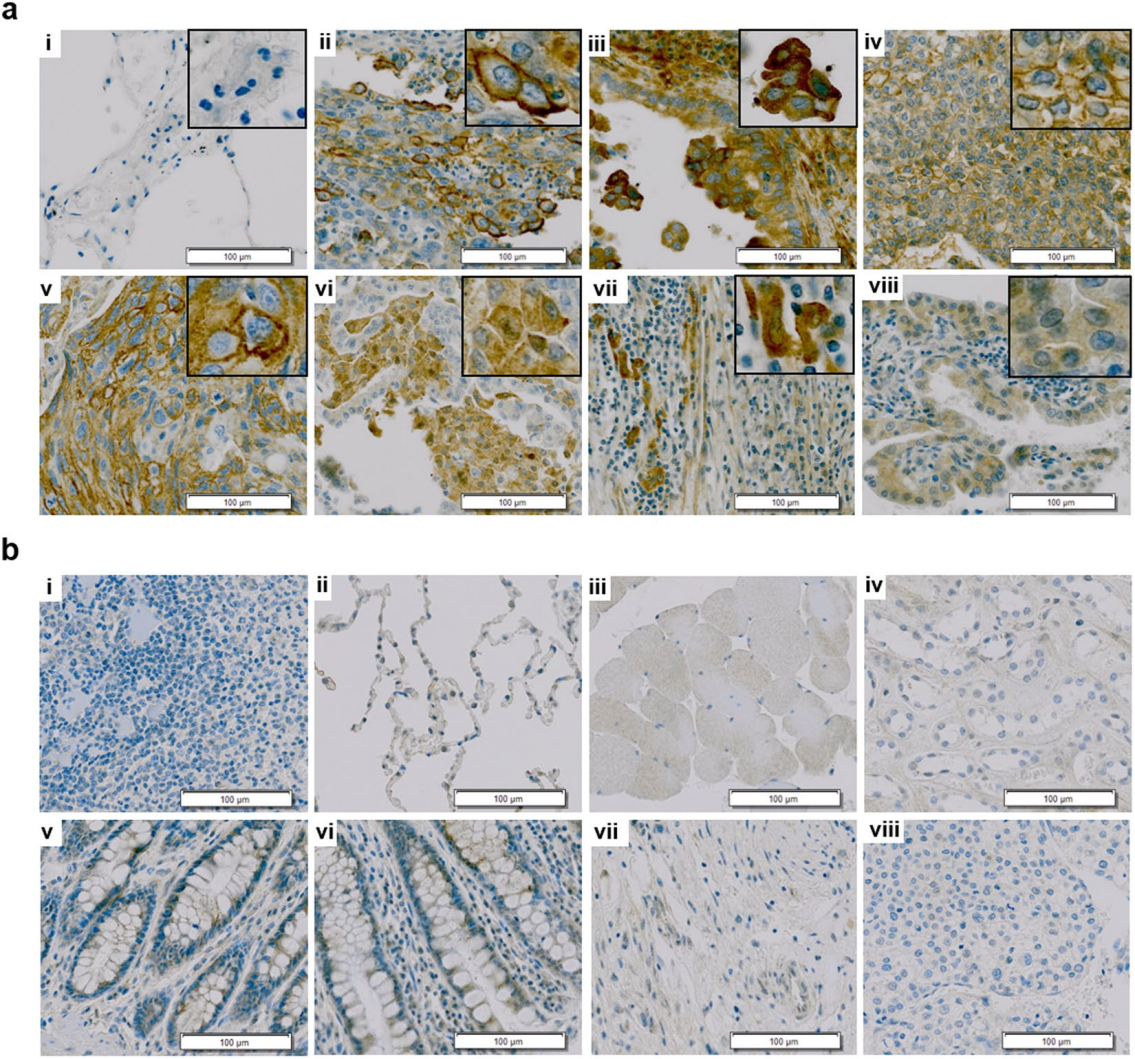
CD55 expression in human lung cancer tissue. (**a**) Immunohistochemical analysis of CD55 in lung tissue. i, normal lung; ii, lung adenosquamous carcinoma; iii, lymph node metastatic carcinoma from lung adenocarcinoma; iv, lung mucoepidermoid carcinoma; v, lung squamous cell; vi, lung adenocarcinoma; vii, lung large cell carcinoma; viii, lung bronchioloalveolar carcinoma. Scale bars = 100 μm. Insets are magnified images demonstrating CD55 expression on the cell membrane and in the cytoplasm. (**b**) Immunohistochemical analysis of CD55 expression in normal organs. i, spleen; ii, lung; iii, skeletal muscle; iv, kidney; v, rectum; vi, colon; vii, stomach; viii, liver. Scale bars = 100 μm.

We investigated whether CD55 was differentially expressed in lung cancer compared to normal tissue using immunohistochemistry. CD55 was expressed in 68.89% (31/45) of lung cancer tissue specimens (Table 1). Of the 34 NSCLC specimens, 26 (76.47%) were positive for CD55 (Table 1 and Supplementary Table S1). It was predominantly expressed on cell membranes (Fig. 1a,ii, iii,iv, and v, insets) and in the cytoplasm in NSCLC tissue (Fig. 1a,vi,vii, and viii, insets). In contrast, little to no CD55 expression was observed in normal tissue (Fig. 1b). These results suggested that CD55 could be a promising target of an immunotherapy in NSCLC.

**Table 1:** CD55 expression in human lung cancer tissue.

Lung cancer type	Number of stained tissues			Total number of positive tissues
	Strong	Moderate	Negative	
Cancer	18/45	13/45	14/45	31/45 (68.89%)
NSCLC	14/34	12/34	8/34	26/34 (76.47%)
SCLC	0/2	0/2	2/2	0/2
Metastatic	4/9	1/9	4/9	5/9
Normal	1/10	2/10	7/10	3/10

Immunohistochemical analysis of CD55 in lung cancer compared to normal tissue. Strong, moderate, and negative indicate >50%, 10–50%, and <10% positivity, respectively.

### Development of a novel chimeric anti-CD55-specific monoclonal antibody

**Figure 2:**
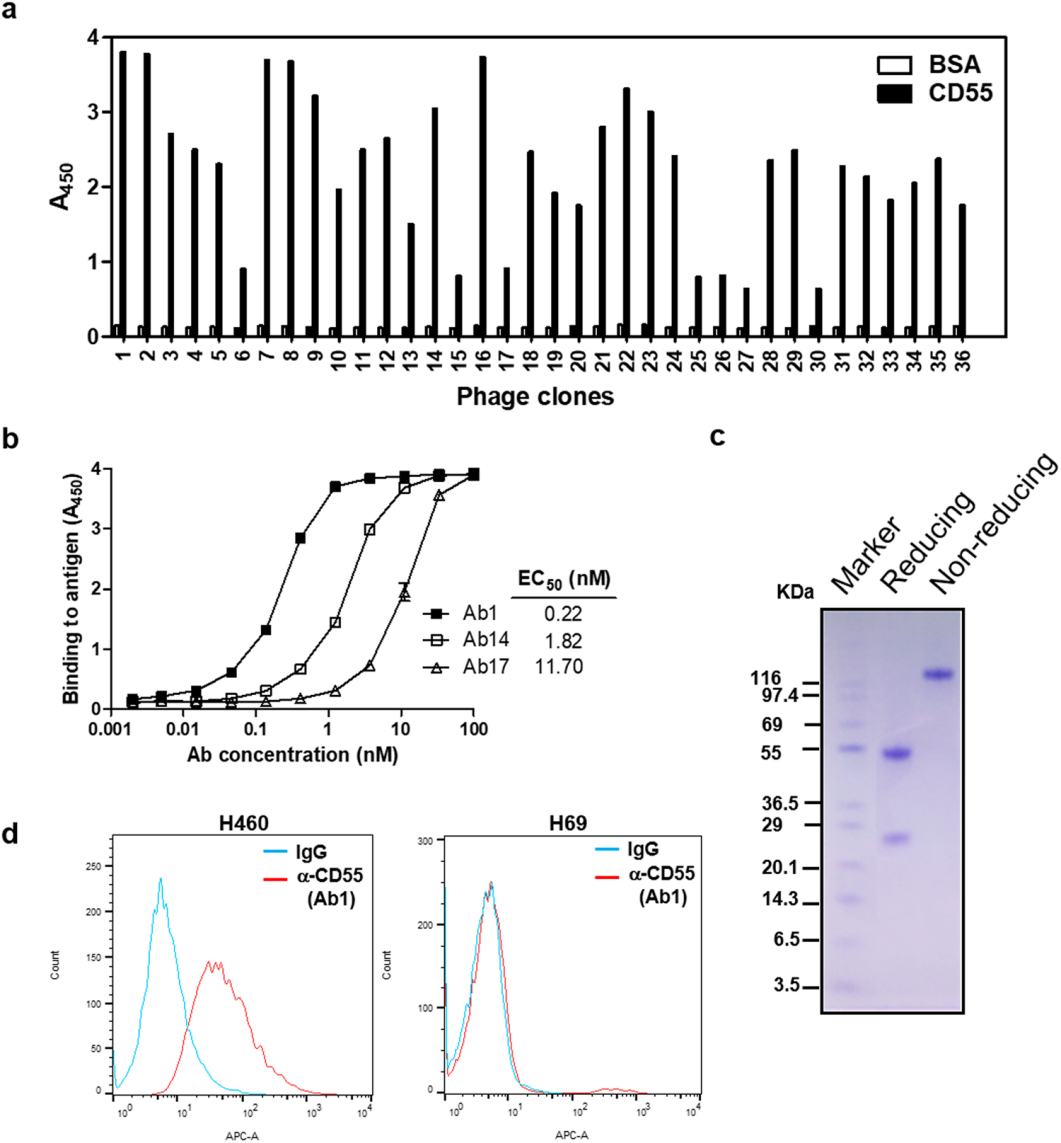
Development of chimeric CD55-specific monoclonal antibodies. (**a**) Phage ELISA results for individual clones tested against recombinant human CD55. A total of 384 clones were tested for binding to recombinant human CD55 (Supplementary Fig. 1). The data for 36 clones are shown. (**b**) Reactivity of three anti-CD55 IgGs to recombinant human CD55 analyzed by antigen-binding ELISA. (**c**) SDS-PAGE analysis of the Ab1 anti-CD55 IgG antibody. (**d**) Representative flow cytometry analysis of H460 and H69 cells stained with the Ab1 anti-CD55 antibody.

We next developed a novel chimeric anti-CD55-specific monoclonal antibody constructed from phage-displayed antibody fragments. A naïve chicken phage-displayed scFv library was biopanned with recombinant human CD55-coated magnetic beads. After four rounds of biopanning, 384 individual clones were selected. Binding reactivity was analyzed by phage enzyme-linked immunosorbent assay (ELISA) (Fig. 2a and Supplementary Fig. S1). Three of the clones (Ab1, Ab14, and Ab17) were isolated repeatedly and contained distinct HCDR3 sequences. The selected scFv clones were then converted to the IgG form. The variable region of either the heavy (VH) or light chain (VL) of each clone was combined with the human constant region of the heavy (CH1-3) and light chain (Ckappa), respectively, using PCR. The chicken/human chimeric antibody constructs were cloned into a mammalian expression vector and transfected into HEK293F cells for production. The purified IgG clones showed the same binding profiles as in the phage ELISA. Ab1 demonstrated higher CD55 binding affinity than Ab14 and Ab17 (Fig. 2b). The Ab1 clones demonstrated >99% purity (Fig. 2c). The specificity of the Ab1 anti-CD55 monoclonal antibody was confirmed by flow cytometry analysis of CD55-positive H460 cells (NSCLC derived from pleural effusions) and CD55-negative H69 cells (small cell lung carcinoma) (Fig. 2d). CD55 expression in lung cancer cell lines was validated by immunoblotting with an anti-CD55 antibody (ab54595; Abcam; Supplementary Fig. S2).

### Characterization of the ^177^Lu-DTPA-anti-CD55 antibody *in vitro*

**Figure 3:**
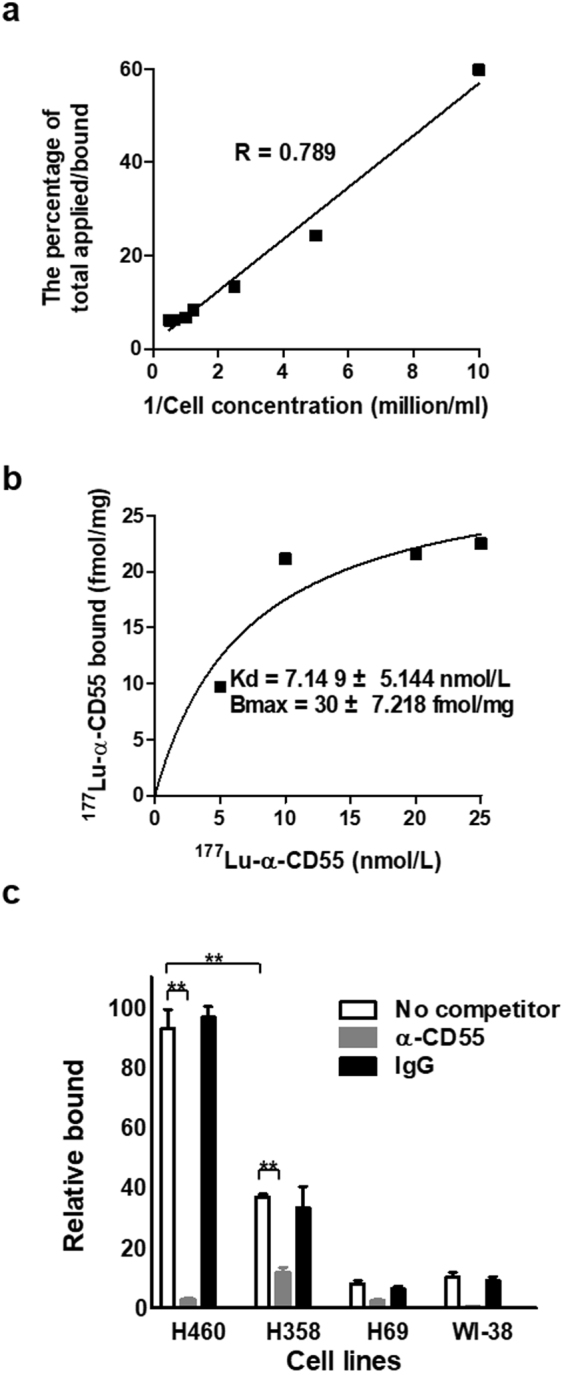
Characterization of ^177^Lu-DTPA-anti-CD55 *in vitro*. (**a**) Representative results of three independent Lindmo assays to examine binding of ^177^Lu-anti-CD55 to H460 cells. (**b**) Representative results of three independent saturation binding assays of ^177^Lu-anti-CD55 in H460 cells. (**c**) Blocking assays in H460, H358, H69, and WI-38 cells (n = 3; **P < 0.01; Student’s t test). The results are presented as the mean ± standard error of the mean (SEM; error bars).

We radiolabeled the Ab1 anti-CD55 monoclonal antibody with ^177^Lu via DTPA and achieved >98% radiochemical purity (Supplementary Fig. S3). The Ab1 ^177^Lu-DTPA-anti-CD55 antibody (^177^Lu-anti-CD55) was stable in serum *in vitro* (Supplementary Fig. S4). Since free ^177^Lu can deposit in bone^27^, stability of ^177^Lu-anti-CD55 adds less hematological toxicity. Lindmo cell-binding assays demonstrated an immunoreactive fraction of 78.9 ± 5.4% (Fig. 3a). Additionally, ^177^Lu-anti-CD55 bound with high affinity to H460 cells, with a Kd of 7.149 ± 5.144 nmol/L and a Bmax of 30 ± 7.218 fmol/mg (Fig. 3b). The capacity of CD55 to bind to ^177^Lu-anti-CD55 antibody was confirmed by competitive binding assays using H460 lung cancer cells with high CD55 expression (CD55^high^), H358 cells with moderate CD55 expression (CD55^mod^), and H69 cells with low CD55 expression (CD55^low^), as well as WI-38 normal lung cells with low CD55 expression (CD55^low^). These results indicated that the binding of the ^177^Lu-anti-CD55 antibody to H460 and H358 cells was CD55-specific (Fig. 3c). The binding specificity of the ^177^Lu-anti-CD55 antibody was demonstrated by competition assays with an unlabeled anti-CD55 antibody (Fig. 3c). Following binding and internalization of ^177^Lu-anti-CD55, a slow release of radioactivity was observed (Supplementary Fig. S5). These results demonstrated that the purity, stability, specific binding activity, and influx-efflux kinetics of the anti-CD55 monoclonal antibody were retained after labeling with ^177^Lu.

### Characterization of the ^177^Lu-anti-CD55 antibody in a mouse model of pleural metastatic lung cancer

We next investigated whether ^177^Lu-anti-CD55 selectively targeted pleural metastatic lung tumors in mice. Injection of H460 cells directly into the pleural cavity resulted in the development of pleural metastatic lung cancer 10 days post-injection (p.i.) (Supplementary Fig. S6). Pleural metastatic tumors were observed infiltrating the neighboring lung, chest wall, and bone. Tumors were positive for CD55 expression by immunohistochemistry (Supplementary Fig. S7). We evaluated CD55 expression in normal organs of balb/c mice to minimize adverse effects before treating the mice with ^177^Lu-anti-CD55. CD55 expression was not detected in normal tissues as expected (Supplementary Fig. S8).

**Figure 4:**
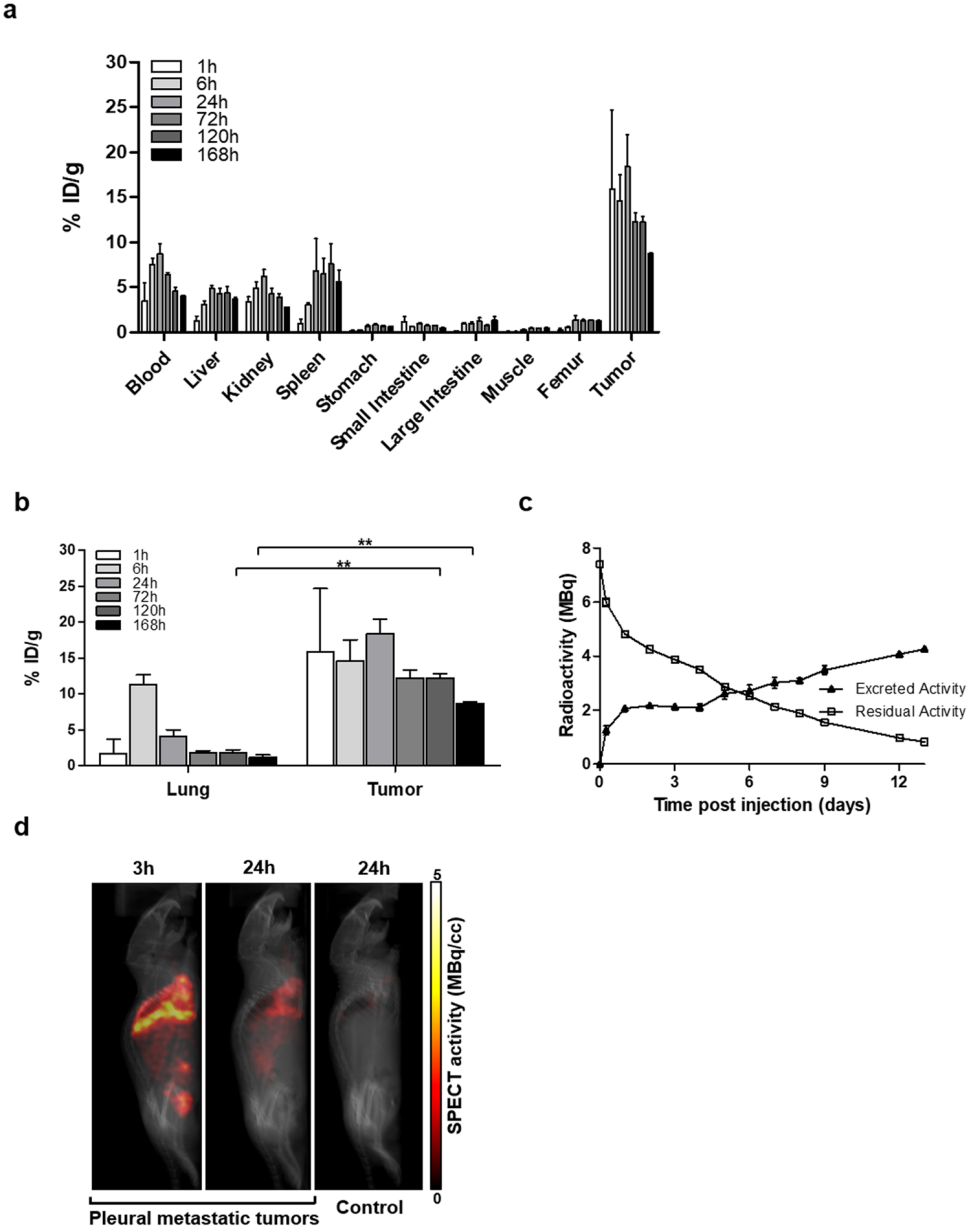
Biodistribution analysis of ^177^Lu-anti-CD55 *in vivo*. (**a**) The biodistribution of ^177^Lu-anti-CD55 in tissue from pleural metastatic mice (n = 3–6 per condition). The results are presented as the mean ± SD (error bars). (**b**) The biodistribution of ^177^Lu-anti-CD55 in normal lung and lung tumor tissue from pleural metastatic mice (n = 3–6 per condition; **P < 0.01; Student’s t test). The results are presented as the mean ± SD (error bars). (**c**) Excretion and residual radioactivity after injection of ^177^Lu-anti-CD55 into mice. (**d**) Micro-SPECT/CT images of pleural metastatic or control mice treated with ^177^Lu-anti-CD55. The amount of radioactivity is calculated in MBq/cc.

We evaluated the biodistribution (tumor uptake and intratumoral distribution) of the ^177^Lu-anti-CD55 antibody *in vivo* (Fig. 4a and Table 2). The ^177^Lu-anti-CD55 antibody was predominantly retained in tumor tissues at all time points. The accumulation peaked in tumors to 18.35 ± 3.58% ID (% of initial dose)/g after 24 hours. Importantly, ^177^Lu-anti-CD55 accumulated in pleural metastatic tumors but not in normal lung tissue (Fig. 4b). We compared the biodistribution and tumor uptake of ^177^Lu-anti-CD55 to the non-selective ^177^Lu-IgG antibody (Supplementary Fig. S9). The highest levels of the ^177^Lu-anti-CD55 antibody in normal organs were observed in blood samples (8.70 ± 1.09% ID/g at 24 hours). The levels gradually cleared to 1.49% ID/g by approximately 18 days, with a half-life of 183 hours (Supplementary Fig. S10). The total residual radioactivity gradually decreased as the antibody was cleared (Fig. 4c). The levels of ^177^Lu-anti-CD55 in tumor tissues were 1.9–4.5 times higher than those in blood (Supplementary Fig. S11 and Table 2) and 19.7–199 times higher than those in muscle (Table 2).

**Table 2:** Biodistribution of the ^177^Lu-anti-CD55 antibody in a pleural metastatic mouse model.

Organ	1 hr	6 hr	24 hr	72 hr	120 hr	168 hr
Blood	3.52 ± 1.94	7.48 ± 1.25	8.70 ± 1.09	6.43 ± 0.36	4.5 ± 0.80	4.00 ± 0.18
Liver	1.24 ± 0.54	3.06 ± 0.77	4.84 ± 0.31	4.30 ± 0.95	4.35 ± 1.2	3.68 ± 0.30
Kidney	3.38 ± 0.57	4.89 ± 1.23	6.19 ± 0.78	4.24 ± 1.02	3.91 ± 0.60	2.79 ± 0.04
Spleen	0.92 ± 0.55	3.03 ± 0.34	6.83 ± 3.58	6.46 ± 2.97	7.57 ± 3.91	5.58 ± 1.83
Stomach	0.12 ± 0.09	0.25 ± 0.05	0.64 ± 0.17	0.86 ± 0.19	0.68 ± 0.16	0.62 ± 0.10
Small Intestine	1.15 ± 0.55	0.60 ± 0.05	0.91 ± 0.14	0.73 ± 0.17	0.72 ± 0.12	0.49 ± 0.02
Large Intestine	0.11 ± 0.04	0.92 ± 0.24	0.97 ± 0.22	1.21 ± 0.69	0.72 ± 0.14	1.32 ± 0.59
Muscle	0.08 ± 0.05	0.10 ± 0.04	0.30 ± 0.03	0.47 ± 0.06	0.43 ± 0.11	0.44 ± 0.14
Femur	0.25 ± 0.17	0.59 ± 0.04	1.39 ± 0.51	1.22 ± 0.32	1.32 ± 0.12	1.22 ± 0.18
Tumor	15.92 ± 8.75	14.59 ± 5.10	18.35 ± 3.58	12.22 ± 1.54	12.22 ± 1.10	8.70 ± 0.20
T/B	4.52	1.95	2.11	1.90	2.72	2.18
T/M	199.00	145.90	61.17	26.00	26.00	19.77
T/L	12.84	4.77	3.79	2.84	2.81	2.36
T/K	4.71	2.98	2.96	2.88	3.13	3.12

Results are expressed as % ID/g ± SD (n = 3). T/B, tumor to blood ratio; T/M, tumor to muscle ratio; T/L, tumor to liver ratio; T/K, tumor to kidney ratio.

Micro-single photon emission computed tomography/computed tomography (SPECT/CT) imaging demonstrated high uptake of ^177^Lu-anti-CD55 in H460-derived tumors in pleural metastatic mice (Fig. 4d). Radioactivity was highest in the pleural cavity but was also observed in the spleen and bladder. Clearance of ^177^Lu-anti-CD55 from non-CD55-expressing tissues was observed 24 hours p.i. (Fig. 4d, second panel). Reduced uptake of ^177^Lu-anti-CD55 was observed in the control group (Fig. 4d, third panel) compared to the metastatic lung cancer group (4.2% vs. 15.3% ID, respectively) after 24 hours (Fig. 4d, second panel). These results demonstrated that ^177^Lu-anti-CD55 specifically targeted CD55-expressing metastatic lung cancer cells.

### The ^177^Lu-anti-CD55 antibody inhibits lung cancer cell invasion and migration *in vitro*

**Figure 5:**
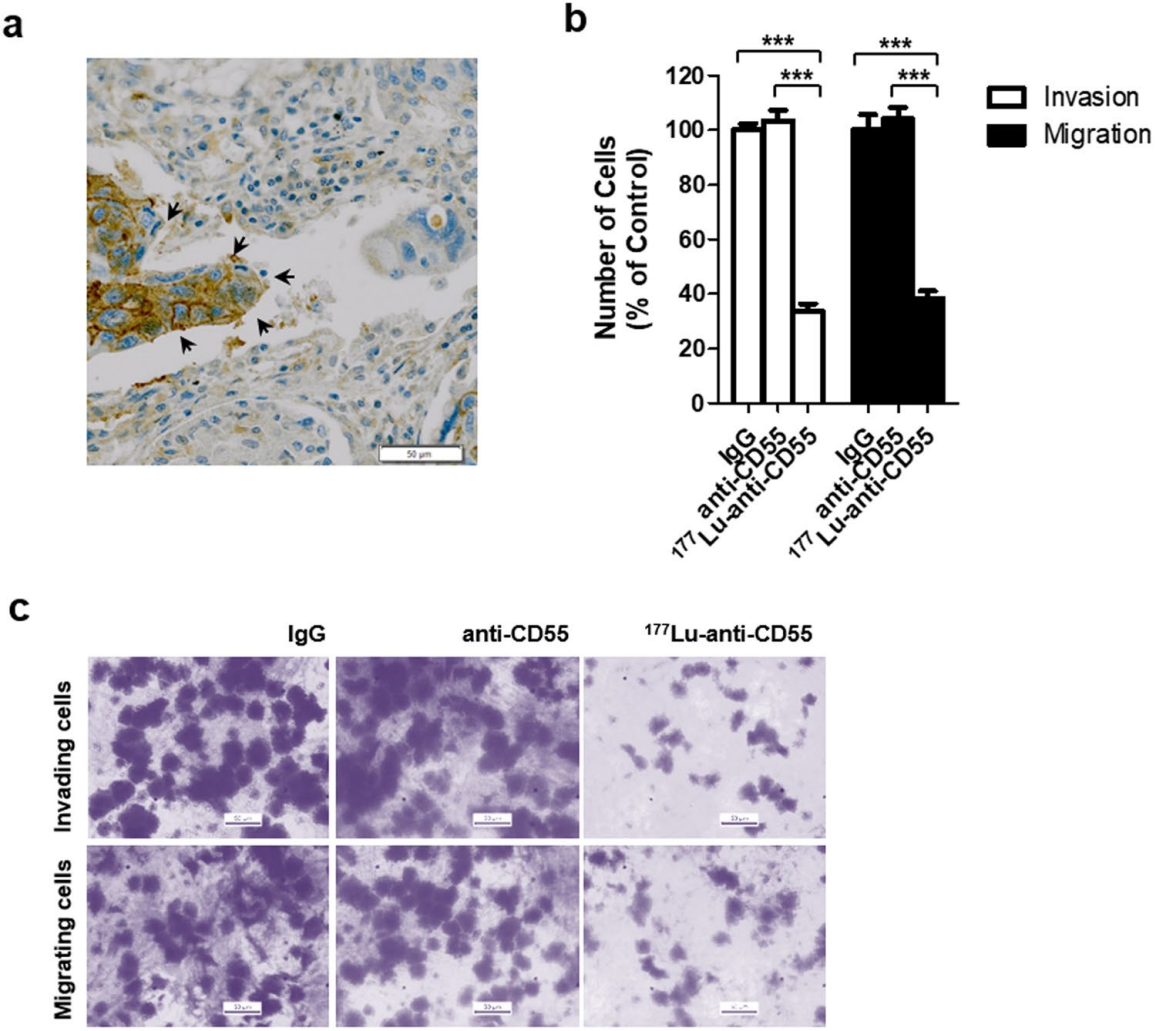
Effects of the ^177^Lu-anti-CD55 antibody on invasion and migration in lung cancer cells. (**a**) The invasive front of squamous cell carcinoma in the lung (arrows). Scale bars = 50 μm. (**b**) Invasion and migration of H460 cells treated with IgG, anti-CD55, or ^177^Lu-anti-CD55. (**c**) Invasion and migration were quantified by counting cells in ten randomly selected regions (***P < 0.001; Student’s t test). The results are presented as the means ± SEM (error bars).

High CD55 expression was observed in metastatic tumor cells (80%, 4/5) compared to NSCLC cells (53.85%, 14/26) among CD55-positive cases (Table 1). CD55 was highly expressed at the invading front of the tumor in lung squamous cell carcinoma (Fig. 5a). Therefore, we examined whether ^177^Lu-anti-CD55 inhibited lung cancer cell invasion and migration *in vitro*. Treatment of H460 cells with ^177^Lu-anti-CD55 resulted in a 66.23% reduction in lung cancer cell invasion and a 61.51% reduction in migration (Fig. 5b,c). These results suggested that ^177^Lu-anti-CD55 could block metastasis of lung cancer cells. However, we did not exclude the possibility that ^177^Lu-anti-CD55 inhibited metastasis by decreasing tumor cell viability.

### Characterization of the ^177^Lu-anti-CD55 antibody in lung cancer cells and in a mouse model of pleural metastatic lung cancer

**Figure 6:**
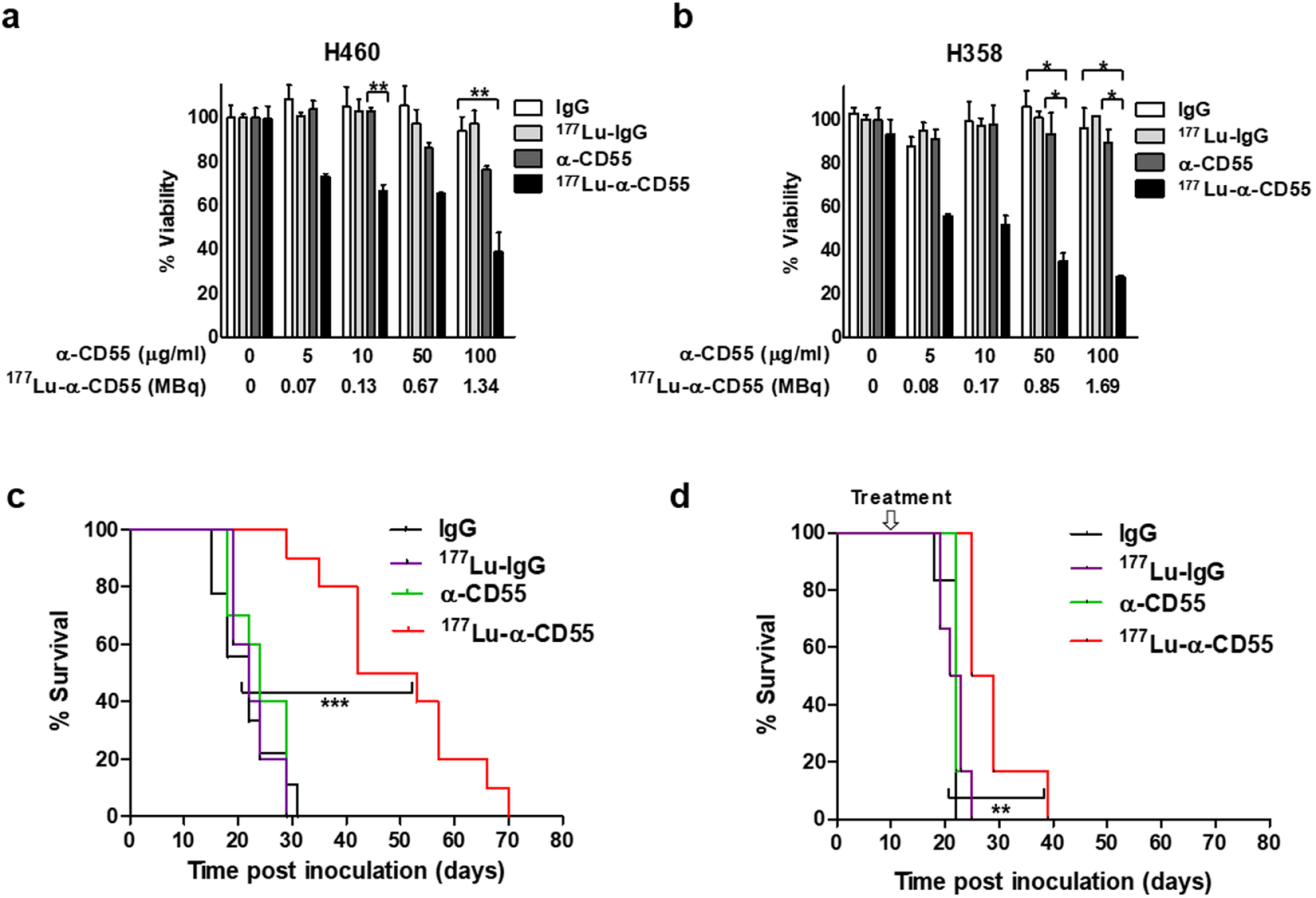
Therapeutic efficacy of the ^177^Lu-anti-CD55 antibody *in vitro* and *in vivo*. (**a**) Assays of H460 lung cancer cell viability after treatment with IgG, ^177^Lu-IgG, anti-CD55, or ^177^Lu-anti-CD55. α-CD55 (μg/ml) indicates the concentration of all the antibodies, IgG, ^177^Lu-IgG, α-CD55, and ^177^Lu-α-CD55. ^177^Lu-α-CD55 (MBq) indicates the corresponding amounts of radioactivity of ^177^Lu-α-CD55 for the indicated concentration (n = 3; **P < 0.01; Student’s t test). The results are presented as the mean ± SEM (error bars). (**b**) Assays of H358 cell viability after treatment with IgG, ^177^Lu-IgG, anti-CD55, or ^177^Lu-anti-CD55 (n = 3; *P < 0.05; Student’s t test). The results are presented as the mean ± SEM (error bars). (**c**) Effects of ^177^Lu-anti-CD55 on the survival of H460 bearing-early pleural metastatic mice (n = 10 for each group; ***P < 0.001; Log-rank (Mantel-Cox) test). (**d**) Effects of ^177^Lu-anti-CD55 on the survival of pleural metastatic mice (n = 10 for each group; **P < 0.01; Log-rank (Mantel-Cox) test).

Even at low concentrations (5 and 10 µg/ml), where the unlabeled anti-CD55 antibody was ineffective in inducing cell death in H460 cells, ^177^Lu-anti-CD55 reduced cell viability by 26.7% and 33.4%, respectively (Fig. 6a). Additionally, ^177^Lu-anti-CD55 inhibited the survival of H358 cells (bronchioloalveolar carcinoma cells, a subtype of NSCLC), while unlabeled CD55-specific antibodies did not reduce viability (Fig. 6b). H460-induced metastasis in early pleural metastatic mice was attenuated by treatment with 7.4 MBq of ^177^Lu-anti-CD55 (Fig. 6c). Mice treated with ^177^Lu-anti-CD55 demonstrated a 2.15-fold increase in median survival compared to controls (47.5 vs. 22 days, respectively) (Fig. 6c). Similarly, treatment with ^177^Lu-anti-CD55 resulted in a 23% increase in the median survival of pleural metastatic mice compared to controls (27 vs. 22 days, respectively) (Fig. 6d). The ^177^Lu-anti-CD55 antibody was more effective against early pleural metastatic tumors than advanced.

### Cisplatin and ^177^Lu-anti-CD55 have synergistic antitumor effects *in vitro* and *in vivo*

**Figure 7:**
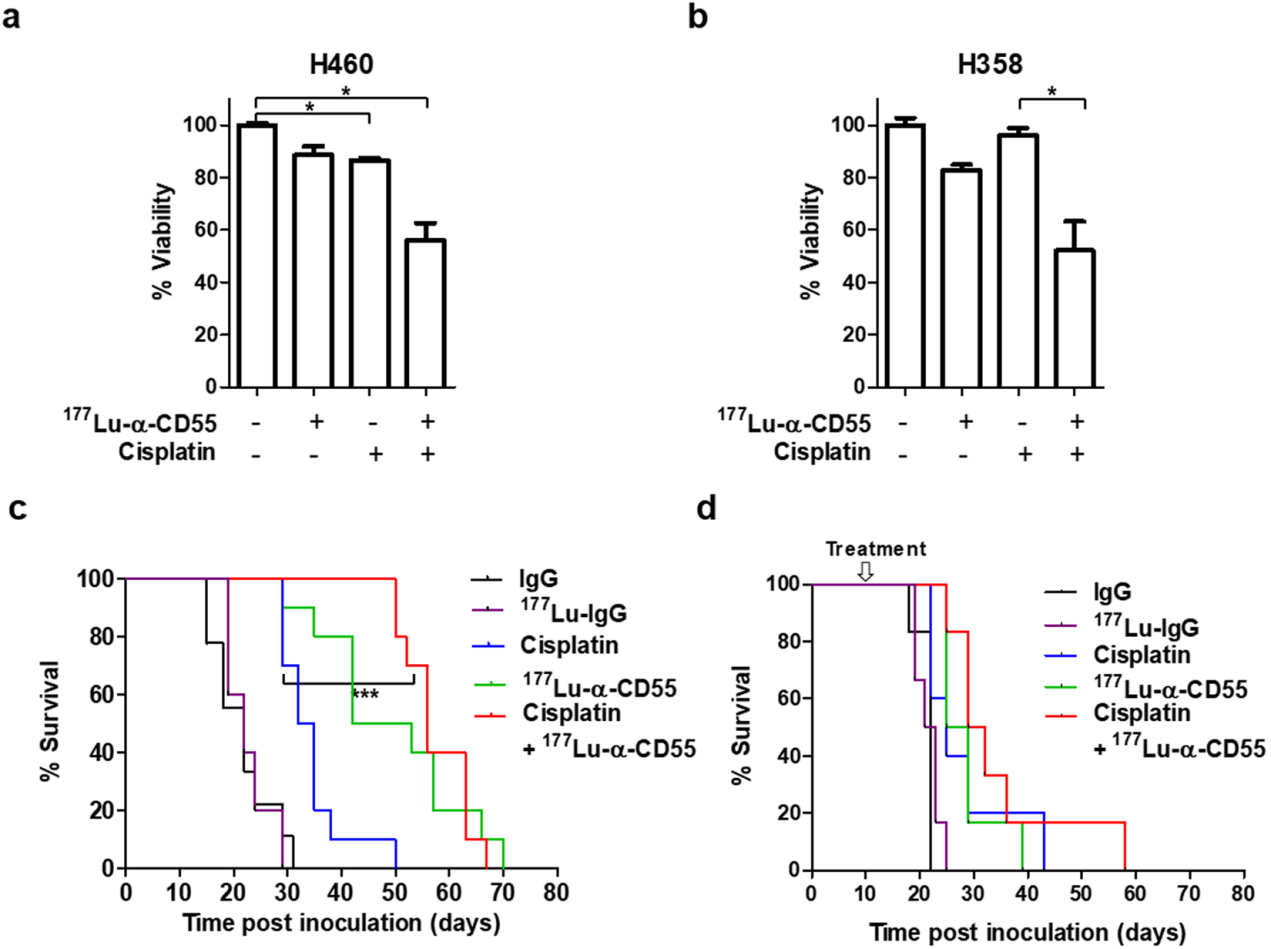
Combinatorial effects of ^177^Lu-anti-CD55 and cisplatin *in vitro* and *in vivo*. (**a**) Cell viability assays of H460 lung cancer cells treated with 0.1 MBq of ^177^Lu-anti-CD55 in the presence or absence of 3 μM cisplatin (n = 3; *P < 0.05; Student’s t test). The results are presented as the mean ± SEM (error bars). (**b**) Cell viability assays of H358 lung cancer cells treated with 0.1 MBq of ^177^Lu-anti-CD55 in the presence or absence of 3 μM cisplatin (n = 3; *P < 0.05; Student’s t test). The results are presented as the mean ± SEM (error bars). (**c**) Effects of ^177^Lu-anti-CD55 in the presence or absence of cisplatin on the survival of early pleural metastatic mice (n = 10 for each group; ***P < 0.001; Log-rank (Mantel-Cox) test). (**d**) Analysis of the survival of pleural metastatic mice after treatment with ^177^Lu-anti-CD55 in the presence or absence of cisplatin (n = 10 for each group).

Cisplatin is widely used for lung cancer treatment^4,28,29^. We hypothesized that ^177^Lu-anti-CD55 could enhance the efficacy of cisplatin since DNA lesions formed by ^177^Lu-anti-CD55 and cisplatin are different—double strand breaks^30^ and cisplatin-DNA adducts^31^. Treatment of H460 cells with either ^177^Lu-anti-CD55 or cisplatin alone reduced cell viability by 11.5% and 13.6%, respectively. Combined treatment of H460 cells with both ^177^Lu-anti-CD55 and cisplatin reduced cell viability by 44.2%, which was indicative of a synergistic effect (Fig. 7a). Additionally, combined treatment of H368 tumor cells with ^177^Lu-anti-CD55 and cisplatin led to a 47.9% reduction in cell viability, which was also indicative of a synergistic effect (Fig. 7b).

Importantly, treatment with ^177^Lu-anti-CD55 was more effective than cisplatin (Fig. 7c), which is a standard treatment choice for patients with pleural metastatic lung cancer. Combined treatment resulted in a 2.54-fold increase in the median survival of early pleural metastatic mice (Fig. 7c) and a 1.39-fold increase in the median survival of advanced pleural metastatic mice compared to controls (56 days, 30.5, and 22 days, respectively) (Fig. 7d).

## Discussion

We demonstrated that ^177^Lu-anti-CD55 has antitumor efficacy in pleural metastatic lung cancer. We selected ^177^Lu, a low energy emitter of both γ- and β-rays, which is favorable for both SPECT imaging and targeted radiotherapy of micrometastases and small tumors. Our data indicate the ^177^Lu labelled anti-CD55 antibody is functionally equivalent to the unlabeled anti-CD55 antibody, suggesting that ^68^Ga and ^89^Zr, could also be used for targeted radioimmunotherapy or diagnostic imaging.

The development of a fully humanized anti-CD55 antibody and an optimized protocol such as repeated administration could enhance the efficacy of ^177^Lu-anti-CD55. Interestingly, starvation accelerated the uptake of ^177^Lu-anti-CD55 by up to 10.47-fold (Supplementary Fig. S12). However, additional studies are required to determine whether starvation or caloric restriction could improve the efficacy of ^177^Lu-anti-CD55 *in vivo*.

We focused on whether ^177^Lu-anti-CD55 could improve quality of life in advanced lung cancer patients. First, we showed that the antibody slowed weight loss, which is correlated with quality of life (Supplementary Fig. S13). It may directly attenuate cachexia or act through other factors such as cytokines or neuropeptides^32^. Second, physical isolation for treatment should be reconsidered despite the increase in survival. Thus, we administered a reduced dose of ^177^Lu-anti-CD55 (7.4 MBq, 20 g) to mice compared to that of standard radiopharmaceutical therapies. This dose is equivalent to approximately 1.77 GBq per human (60 kg) based on the United States Food and Drug Administration guidelines^33^. In comparison, 7.4 GBq per human was administered as a clinical standard dose of ^177^Lu-DOTATATE to treat neuroendocrine tumors. Third, we focused on whether ^177^Lu-anti-CD55 demonstrated reduced toxicity. L-lysine and L-arginine can counteract kidney retention of radiopeptides and prevent nephrotoxicity^34^. Although the levels of ^177^Lu-anti-CD55 in the kidney were not high (2.79–6.19% ID/g), we considered the possibility that residual radioactivity could result in toxicity. Additionally, we are currently trying to shorten the half-life of ^177^Lu-anti-CD55 by Ab Fc engineering Chest tube drainage could minimize the absorption of ^177^Lu-anti-CD55 into the blood.

Despite the synergistic antitumor effects of combined cisplatin and ^177^Lu-anti-CD55 treatment *in vitro*, pleural metastatic mice were less susceptible to combined treatment. We speculate that this may be because we evaluated the effects in an advanced lung cancer model. These tumors may not be as susceptible to combined therapy. Given that cisplatin is an effective therapy for early- to advanced-staged lung cancer, combined cisplatin and ^177^Lu-anti-CD55 therapy could be effective for various types of lung cancer.

## Methods

### Immunohistochemistry

Immunohistochemical analysis was performed by SuperBioChips Laboratories as previously described^35^. A 1:200 dilution of the anti-CD55 polyclonal antibody (AP14798A; Abgent) was used for all analyses. Tissue array slides (CCA4E, lung cancer-metastasis-normal; SuperBioChips Laboratories) from multiple lung cancer patients were immunostained and imaged. Formalin-fixed tissue samples from pleural metastatic mice were also analyzed by SuperBioChips Laboratories. The analysis was blinded and the quantification performed by pathologists. The percentage of positive tissues was scored and classified as follows: ≥50% as ‘Strong’, 10–50% as ‘Moderate’, and <10% as ‘Negative”.

### Selection of CD55-specific scFvs using phage display

A naïve chicken phage-displayed scFv library (constructed by SG Medical, Inc. as described previously^36,37^) was used for bio-panning to select CD55-specific scFvs. Briefly, 5 × 10^6^ magnetic beads (Dynabeads M-270 epoxy; Invitrogen) were coated with 2.5 μg of recombinant human CD55 (2009-CD/CF; R&D Systems) for each round of bio-panning (four rounds). After the final round, 384 individual phage clones that displayed scFv were randomly selected from colonies grown on output plates and tested for reactivity to recombinant human CD55 using a phage enzyme immunoassay. ELISA-positive scFv clones were analyzed by DNA sequencing, and three unique scFv clones with different CD55 binding affinities were identified.

### Preparation of anti-CD55 IgG

The variable heavy chain gene of selected scFv clones was amplified using the primers 5′-GCTAGCCGCCACCATGGGCTGGTCCTGCATC ATCCTGTTCCTGGTGGCCACCGCCACCGGCGCCGTGACGTTGGACGAGTCCGGG-3′ and 5′-GGGCCCTTGGTG GAGGCGGAGGAGACGATGACTTCGGTCCC-3′. The variable light chain gene of the clones was amplified using the primers 5′-AAGCTTGCCGCCACCATGGGCTGGTCCTGCATCATCCTGTTCCTGGTGGCCACCGCCACCGGCGCCCTGACTCAGCCGTCCTCGGTG-3′ and 5′-GAGGGGGCGGCC ACGGTCCGTAGGACGGTCAGGGTTGTCCCGGC-3′. The variable heavy chain primers were designed to add NheI and ApaI restriction sites to both the 5′ and 3′ ends. The variable light chain primers were designed to add HindIII and RsrII sites to both the 5′ and 3′ ends. PCR fragments were digested with the appropriate restriction enzymes (NEB) and cloned into the bicistronic mammalian expression vector pCDNA3.1 (Invitrogen), which encodes the hinge and CH2-CH3 domains of human IgG1 downstream of the variable heavy chain cloning site. Anti-CD55 IgGs were produced and purified as described previously^38^.

### Cell culture

H460 (ATCC HTB-177), H358 (ATCC CRL5807), and H69 (ATCC HTB-119) cells were maintained in RPMI-1640 with 10% FBS. WI-38 (ATCC CCL-75) cells were maintained in DMEM with 10% FBS.

Cell survival was quantified using a microplate reader at 450 nm and the Cell Counting kit-8 (Dojindo Molecular Technologies) in the presence of human complement system (S1764; Sigma).

For flow cytometry, the cells were stained with an Alexa Fluor 647-conjugated anti-CD55 antibody (A-20186; Molecular Probes; red histograms) or isotype human control IgG (I4506; Sigma; blue histograms) and analyzed using a BD FACS Canto II.

Invasion and migration assays were performed using a 24-well Transwell system (3422; Costar) as described previously^35^.

### Radiolabeling

The ^177^Lu-anti-CD55 antibody was prepared as previously described^39^ with minor modifications. Briefly, an anti-CD55-specific monoclonal antibody was incubated with a 50-fold molar excess of [(R)-2-Amino-3-(4-isothiocyanatophenyl) propyl]-trans-(S,S)-cyclohexane-1,2-diamine-pentaacetic acid (*p*-SCN-Bn-CHX-A”-DTPA,B-355; Macrocyclics) in 0.1 mol/L NaHCO_3_ buffer (pH 8.2) and conjugated antibodies purified. The *p*-SCN-Bn-CHX-A”-DTPA-conjugated anti-CD55 antibody was labeled with ^177^Lu (Lu-177 n.c.a.; ITG; half-life, 6.71 days) in 0.1 mol/L ammonium acetate buffer (pH 5.4) for 30 min at room temperature.

The immunoreactivity of ^177^Lu-anti-CD55 was evaluated using the Lindmo assay as described previously^40,41^. Briefly, H460 cells (0 to 6.0 × 10^6^) were incubated with 0.074 MBq of ^177^Lu-anti-CD55 for 1 hour. The cells were washed and binding specificity analyzed using Scatchard assays. Radioactivity was quantified using a Wallac 1470 automated gamma counter (PerkinElmer Life Science). Saturation binding analyses were performed as described previously^42^. Blocking assays were performed by plating 1.5 × 10^5^ of the indicated cells in 24-well plates and incubating them with 0.074 MBq of ^177^Lu-anti-CD55 in the presence of 50X unlabeled anti-CD55 antibody. Non-specific binding was quantified as described previously^43^.

### Animal experiments

Animal care and experimental protocols were approved by the Institutional Animal Care and Use Committee at KAERI. All methods were carried out in accordance with relevant guidelines and regulations. For induction of the pleural metastatic mouse model, 1 × 10^7^ H460 cells were suspended in 200 μl of phosphate-buffered saline (the amount of cells was chosen to mimic early pleural metastasis without sudden death of mice within 2 weeks) and injected into the thoracic cavities of male balb/c nude mice. Metastatic tumor nodules were observed 10 days post-injection (p.i). Ten days after injection of H460 cells, mice were injected in the thoracic cavities with ^177^Lu-anti-CD55 (7.4 MBq) or cisplatin (5 mg/kg/week) and survival assessed. For the early pleural metastatic mouse model, each mouse was coinjected in the thoracic and peritoneal cavities with H460 cells and either ^177^Lu-anti-CD55 (7.4 MBq/mouse; 2 mg/kg) or cisplatin (5 mg/kg/week), respectively. Mice were observed daily and body weight measured twice a week. Survival and Kaplan-Meier analyses were performed with GraphPad Prism 5. Log-rank (Mantel-Cox) tests were used to calculate P values for Kaplan-Meier analyses.

### Biodistribution analysis

Biodistribution analysis of ^177^Lu-anti-CD55 in the mice was performed as described previously^42^. The ^177^Lu-anti-CD55 antibody (185 kBq) was injected into the thoracic cavity and the biodistribution assessed at 1, 6, 24, 72, 120, and 168 hours after injection. At each time point, the mice were sacrificed and the relevant organs or tissues excised and weighed. Blood samples were also collected. Counts per minute (cpm) in samples were measured using a Wallac 1470 automated gamma counter (PerkinElmer Life Science). The cumulative activity was calculated as the percent of injected radioactivity dose per gram of tissue (% ID/g). Residual radioactivity was measured with a dose-calibrator and corrected for physical decay from the time of injection. Radioactivity excretion was calculated by subtracting the residual activity from the injected radioactivity.

### Micro-SPECT/CT

The ^177^Lu-anti-CD55 antibody (7.4 MBq) was injected directly into the thoracic cavity of the mice and a 30 min scan was acquired 3 and 24 hours p.i. with a NanoSPECT/CT system (Bioscan) as described previously^42^. Images were acquired using the *In Vivo* Scope software (Bioscan) and PMOD v3.6.

### Data availability

All data generated or analyzed during this study are included in this published article (and its Supplementary Information files).

## Electronic supplementary material

Supplementary Information
