# Supplementary material for: Development of a radionuclide-labeled monoclonal anti-CD55 antibody with theranostic potential in pleural metastatic lung cancer
Source: Sci Rep. 2018 Jun 12;8:8960. doi: 10.1038/s41598-018-27355-8 (PMC5997699; doi:10.1038/s41598-018-27355-8)
Supplement: Supplementary file 1 — Supplementary Information [file 41598_2018_27355_MOESM1_ESM.pdf]

Supplementary Information

Development of a radionuclide-labeled monoclonal anti-CD55 antibody with theranostic potential in pleural metastatic lung cancer

So Hee Dho[1], Soo Yong Kim[1], Chaeuk Chung[2], Eun Ha Cho[1], So-Young Lee[1], Ji Young Kim[3], Lark Kyun Kim[4], Sung-Won Min[5], Jichul Lee[5], Sung Hee Jung[1] & Jae Cheong Lim[1]

[1]Radioisotope Research Division, Department of Research Reactor Utilization, Korea Atomic Energy Research Institute, Daejeon 34057, Republic of Korea.

[2]Division of Pulmonary and Critical Care Medicine, Department of Internal Medicine, Chungnam National University Hospital, Daejeon 35015, Republic of Korea.

[3]Hormone Research Center, School of Biological Sciences and Technology, Chonnam National University, Gwangju 61186, Republic of Korea.

[4]Severance Biomedical Science Institute and BK21 PLUS project to Medical Sciences, Gangnam Severance Hospital, Yonsei University College of Medicine, Seoul 06230, Republic of Korea.

[5]SG Medical, Inc., Seoul 05548, Republic of Korea.

Correspondence:

Jae Cheong Lim
Radioisotope Research Division
Department of Research Reactor Utilization
Korea Atomic Energy Research Institute
Daejeon 34057, Republic of Korea
Phone: +82-42-868-8344
Fax: +82-42-868-8448
email: limjc@kaeri.re.kr

Supplementary Methods

Immunoblotting

For immunoblotting, the following antibodies were used: anti-CD55 (ab54595) from Abcam and anti-tubulin (sc-9104) from Santa Cruz Biotechnology.

Radiochemical purity and stability

The radiochemical purity and stability of ^{177}Lu-anti-CD55 was evaluated using instant thin layer chromatography-silica gel (iTLC-SG) in 0.1 mol/L citrate buffer.

Internalization and externalization assay

Internalization and externalization kinetics of the ^{177}Lu-anti-CD55 antibody were determined as previously described [1].

Supplementary Figures and Tables

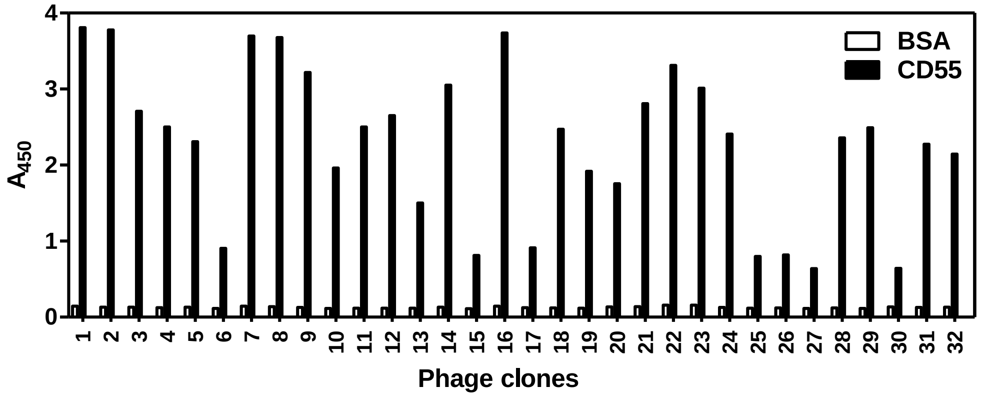

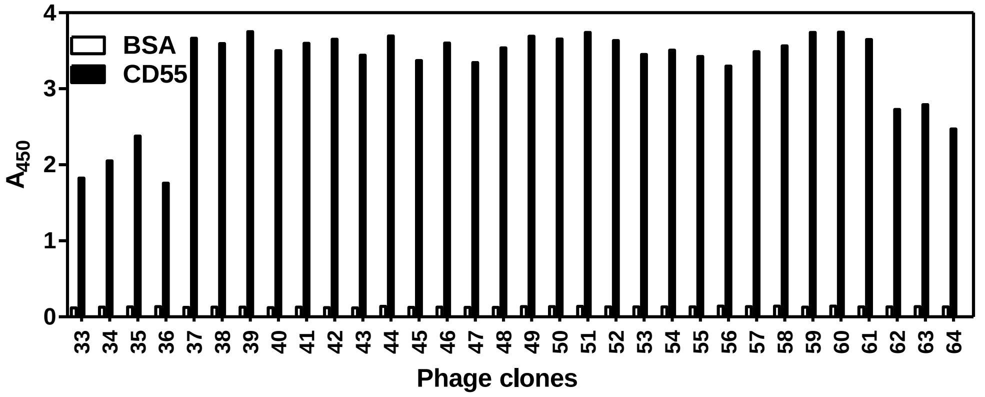

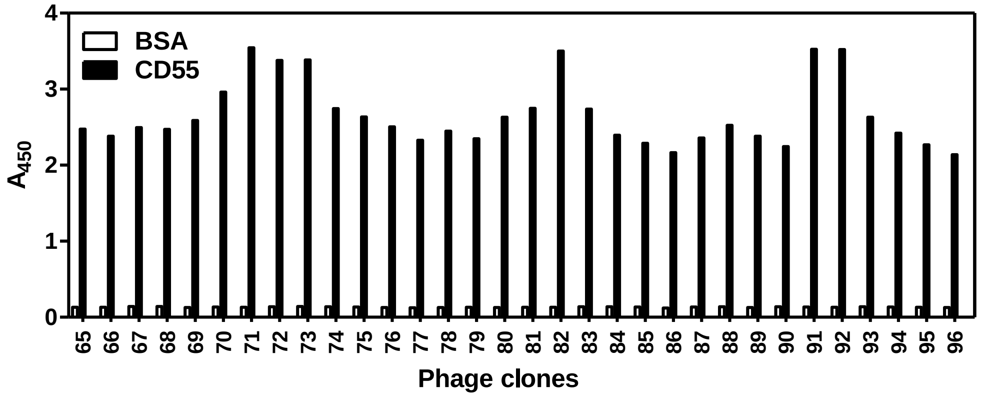

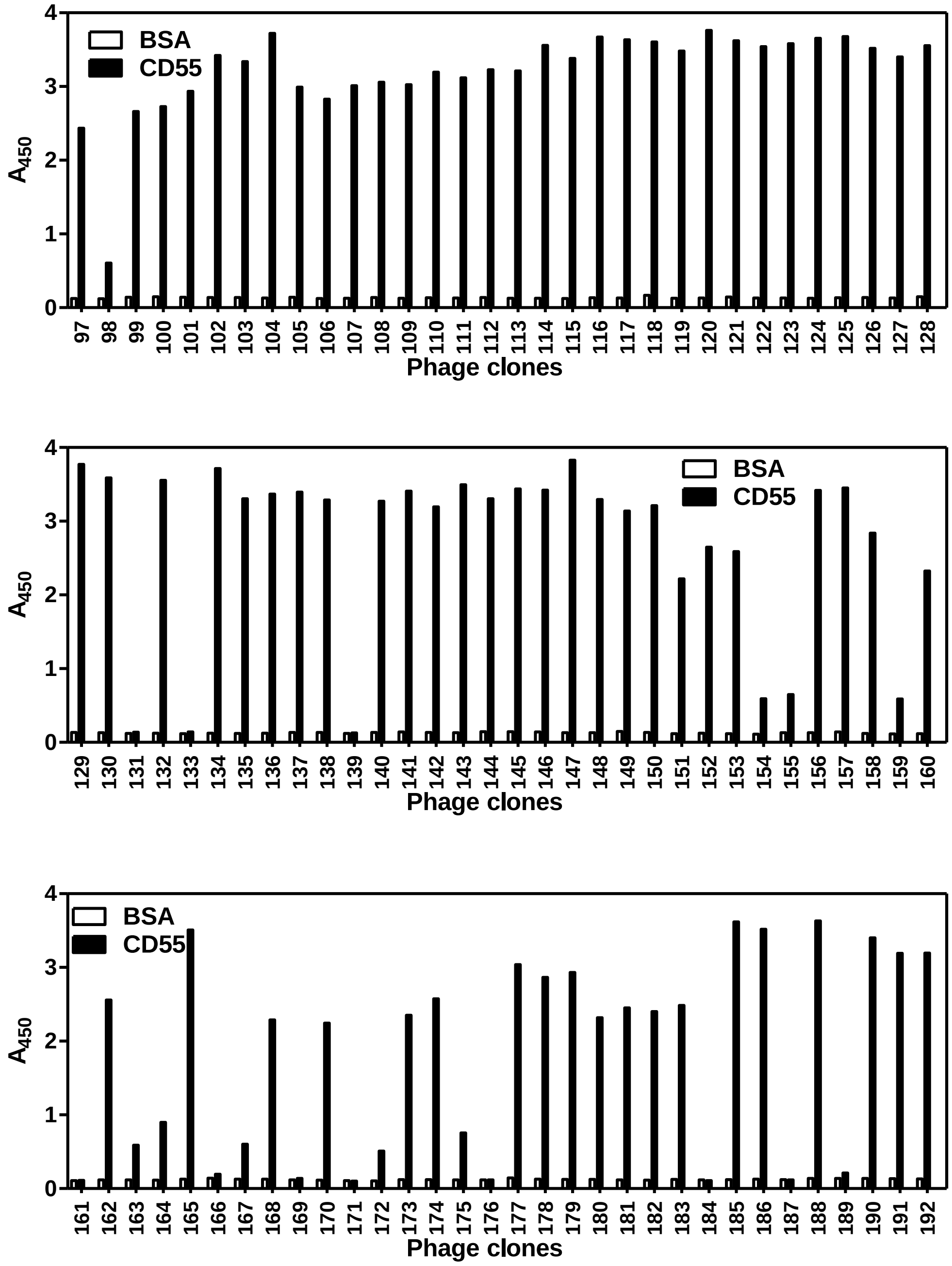

BSA
CD55
A450
0
1
2
3
4
97 98 99 100 101 102 103 104 105 106 107 108 109 110 111 112 113 114 115 116 117 118 119 120 121 122 123 124 125 126 127 128
Phage clones
BSA
CD55
A450
129 130 131 132 133 134 135 136 137 138 139 140 141 142 143 144 145 146 147 148 149 150 151 152 153 154 155 156 157 158 159 160
Phage clones
BSA
CD55
A450
161 162 163 164 165 166 167 168 169 170 171 172 173 174 175 176 177 178 179 180 181 182 183 184 185 186 187 188 189 190 191 192
Phage clones

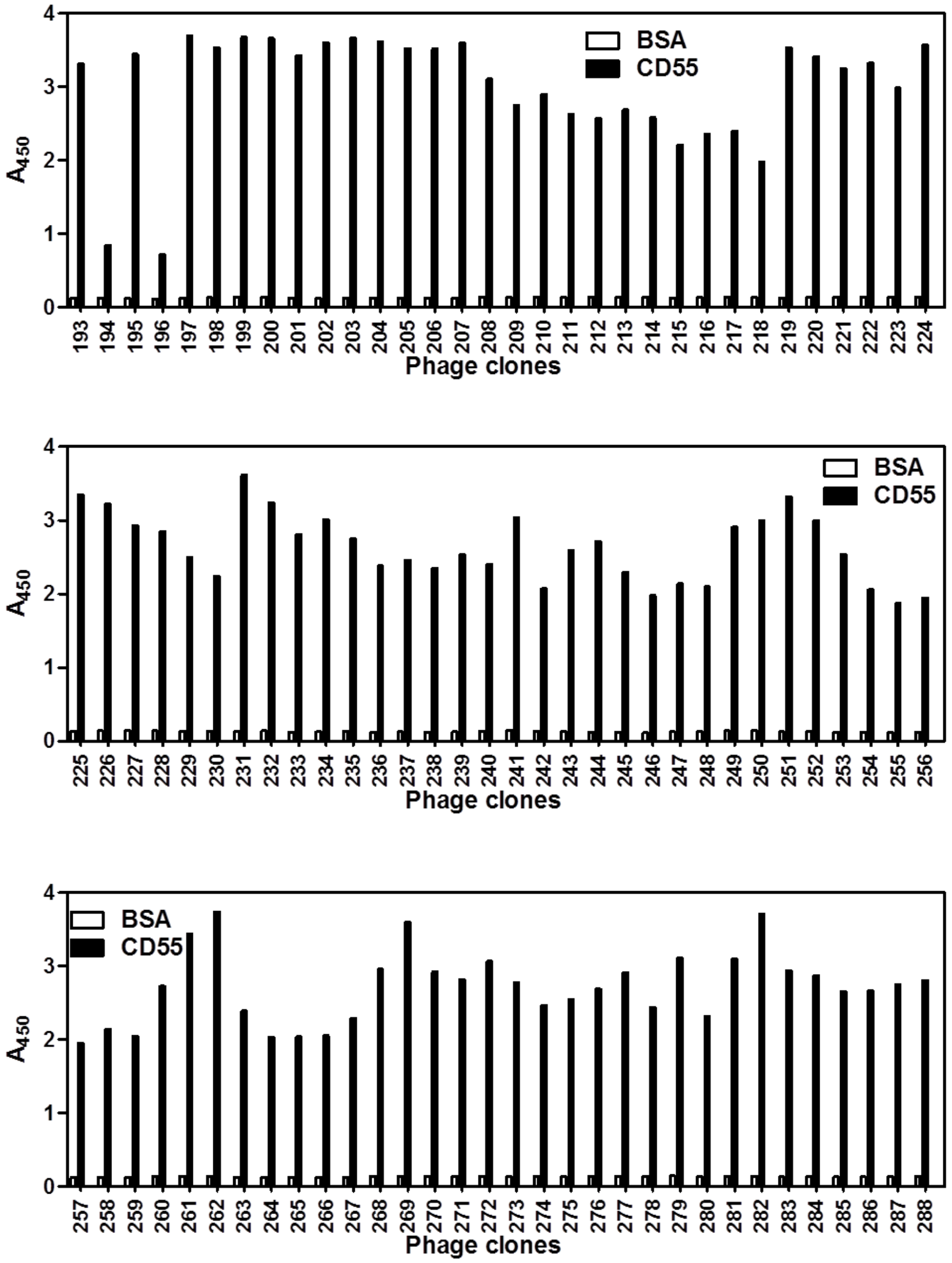
BSA
CD55
A450
0
1
2
3
4
193 194 195 196 197 198 199 200 201 202 203 204 205 206 207 208 209 210 211 212 213 214 215 216 217 218 219 220 221 222 223 224
Phage clones
BSA
CD55
A450
0
1
2
3
4
225 226 227 228 229 230 231 232 233 234 235 236 237 238 239 240 241 242 243 244 245 246 247 248 249 250 251 252 253 254 255 256
Phage clones
BSA
CD55
A450
0
1
2
3
4
257 258 259 260 261 262 263 264 265 266 267 268 269 270 271 272 273 274 275 276 277 278 279 280 281 282 283 284 285 286 287 288
Phage clones

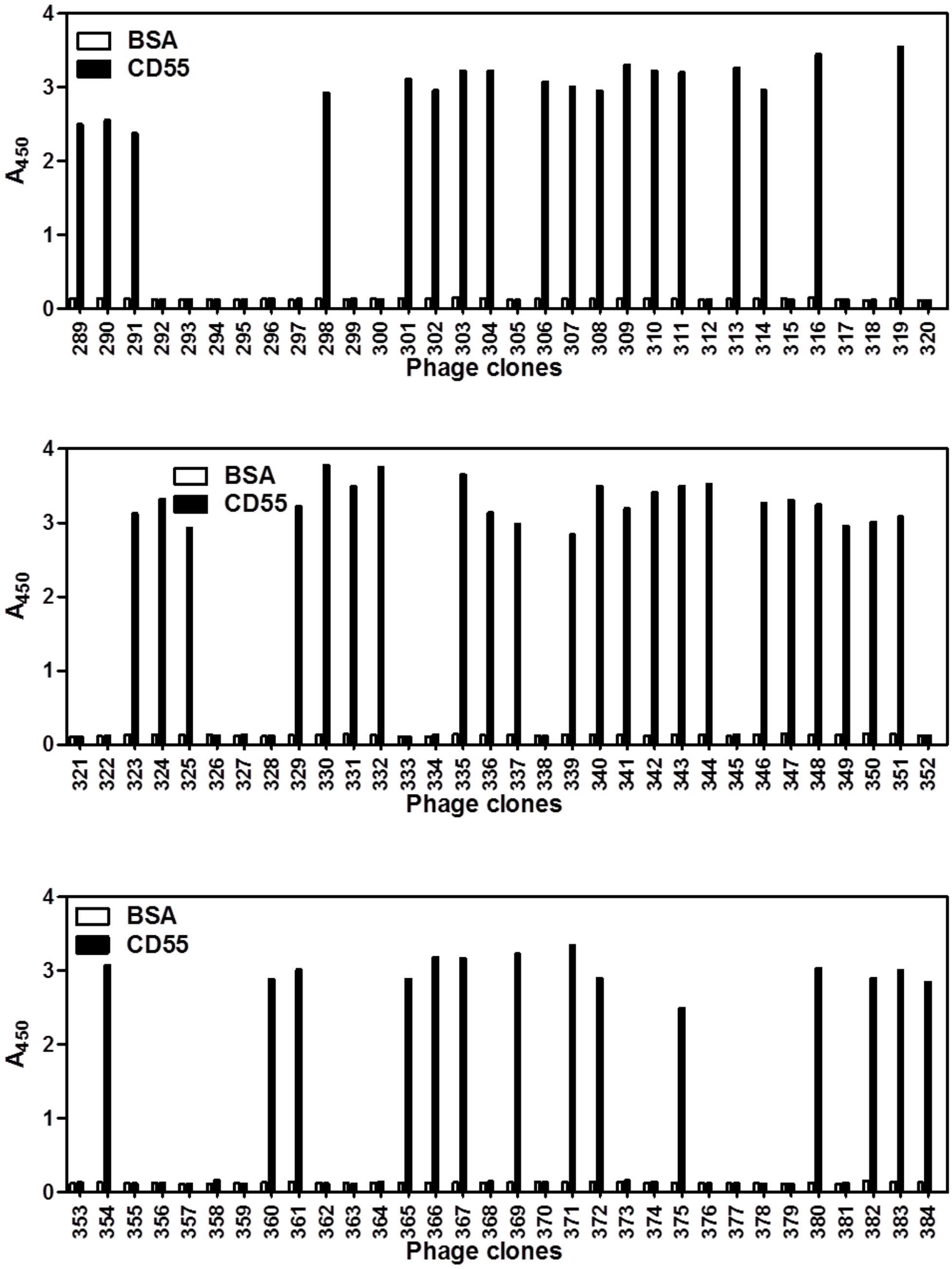

Supplementary Figure S1. Phage ELISA results of individual clones against recombinant human CD55. 384 clones were tested for binding to recombinant human CD55.

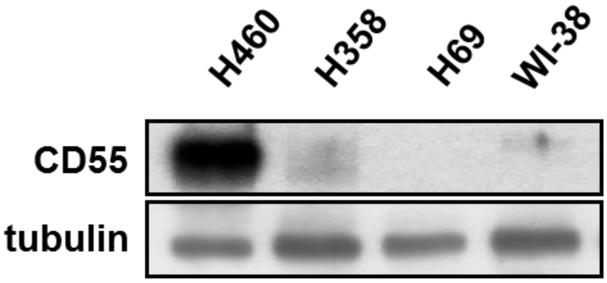

Supplementary Figure S2. The specificity of the Ab1 anti-CD55 monoclonal antibody. Immunoblots of endogenous CD55 and tubulin in H460, H358, H69, and WI-38 cells.

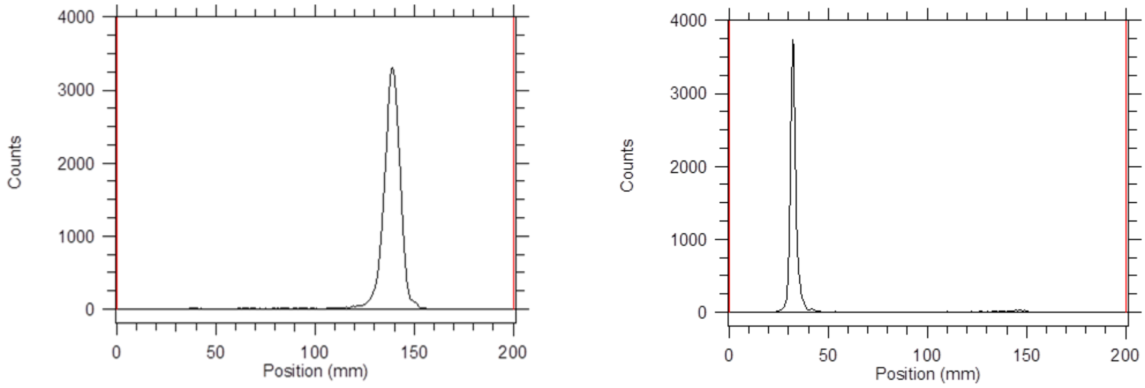

Supplementary Figure S3. Validation of the ^{177}Lu-DTPA-anti-CD55 antibody *in vitro*. Radiochemical purity of ^{177}Lu (left) and ^{177}Lu-anti-CD55 antibody (right) determined by iTLC-SG.

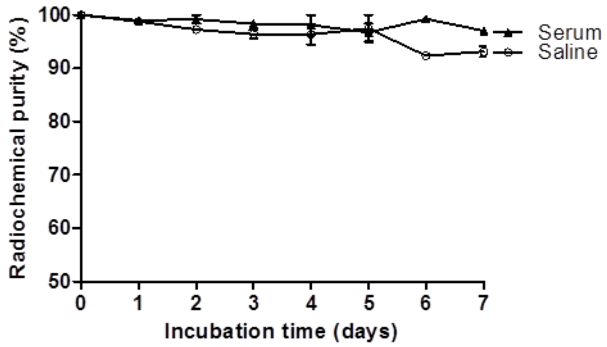

Supplementary Figure S4. Radiochemical stability of the ^{177}Lu-anti-CD55 antibody determined by iTLC-SG. The results are presented as the means ± SEM (error bars).

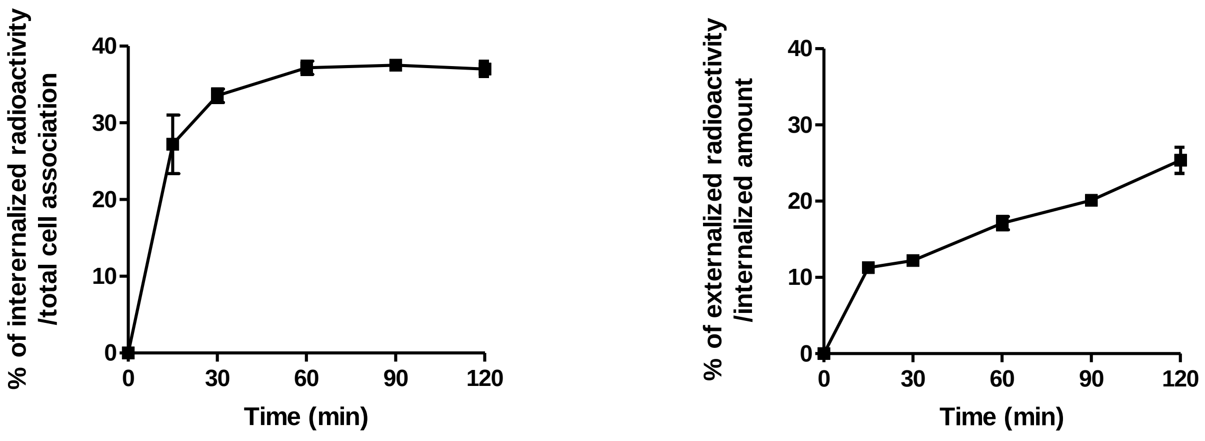

Supplementary Figure S5. Internalization (left) and externalization kinetics (right) of the ^{177}Lu-anti-CD55 antibody in H460 cells. The results are presented as the means ± SEM (error bars).

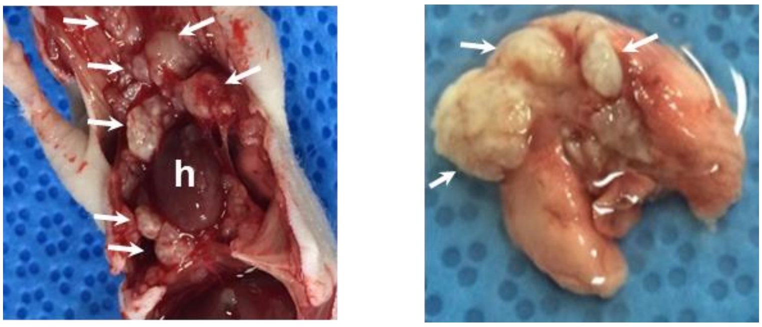

Supplementary Figure S6. Validation of the H460-derived pleural metastatic mouse model. (**left**) Transdiaphragmatic view of the supradiaphragmatic tumor mass (arrows) and heart (h). (**right**) Lung showing multiple tumor foci (arrows).

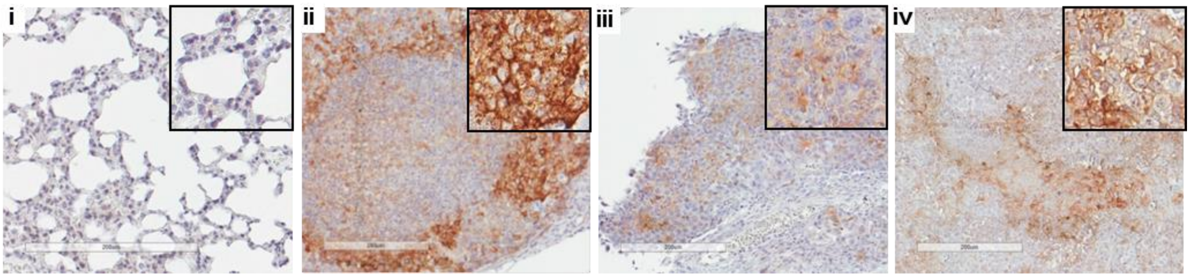

Supplementary Figure S7. Immunohistochemical analysis of CD55 in pleural metastatic mouse tissues. i, normal lung; ii, H460-derived tumor; iii, metastatic tumor infiltrated neighboring lung; iv, metastatic tumor infiltrated neighboring bone. Scale bars = 200 μm.

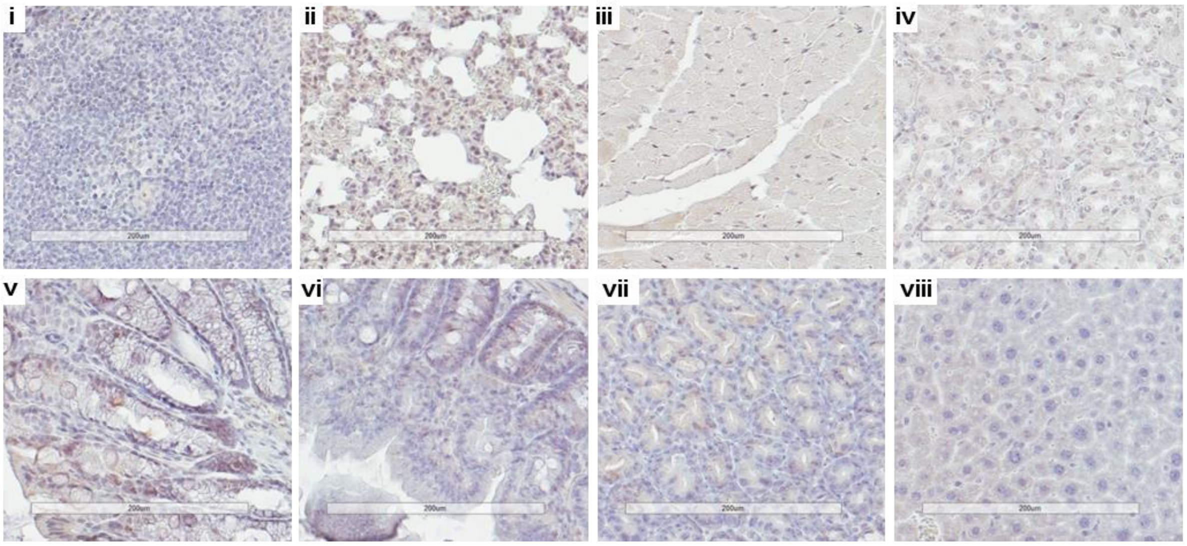

Supplementary Figure S8. Immunohistochemical analysis of CD55 in normal mouse organs. i, spleen; ii, lung; iii, heart; iv, kidney; v, small intestine; vi, colon; vii, stomach; viii, liver. Scale bars = 200 μm.

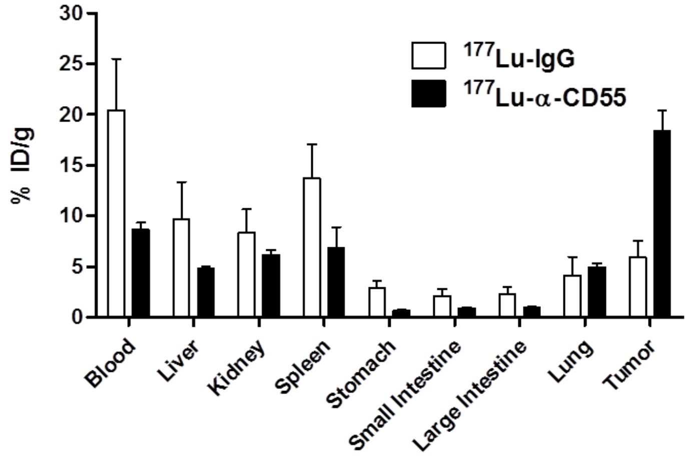

Supplementary Figure S9. The biodistribution of the ^{177}Lu-IgG and ^{177}Lu-anti-CD55 antibody in tissues of pleural metastatic mice (n = 3-6 per condition) at 24h. The results are presented as the means ± SD (error bars).

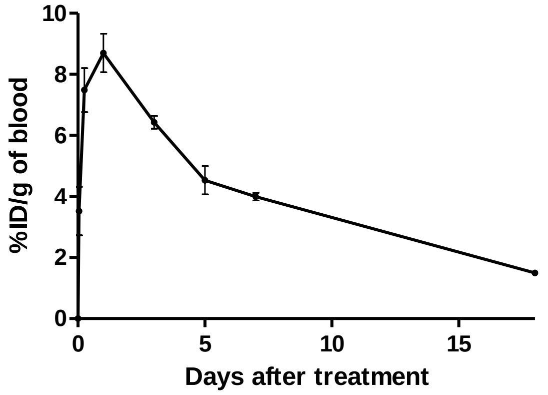

Supplementary Figure S10. The biodistribution of the ^{177}Lu-anti-CD55 antibody in blood.

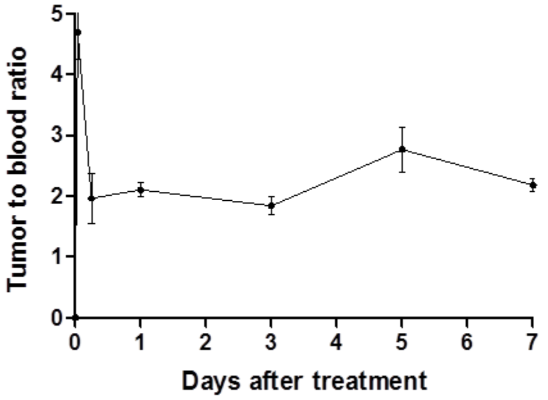

Supplementary Figure S11. Tumor to blood ratio of the ^{177}Lu-anti-CD55 antibody.

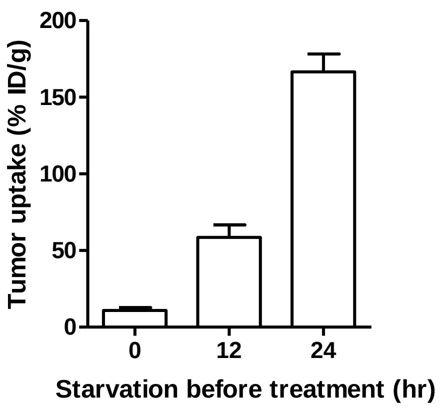

Supplementary Figure S12. The effect of starvation on the uptake of the ^{177}Lu-anti-CD55 antibody in tumors.

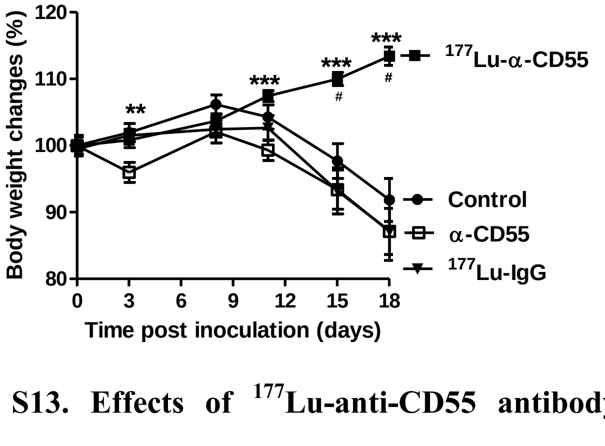

Supplementary Figure S13. Effects of ^{177}Lu-anti-CD55 antibody on body weight changes (%) in mice with H460 bearing-metastatic mice (n = 7-10 per condition; $^{**}P < 0.01$, $^{***}P < 0.001$ vs. α-CD55; $^{\#}P < 0.05$ vs. control; Student's t test).

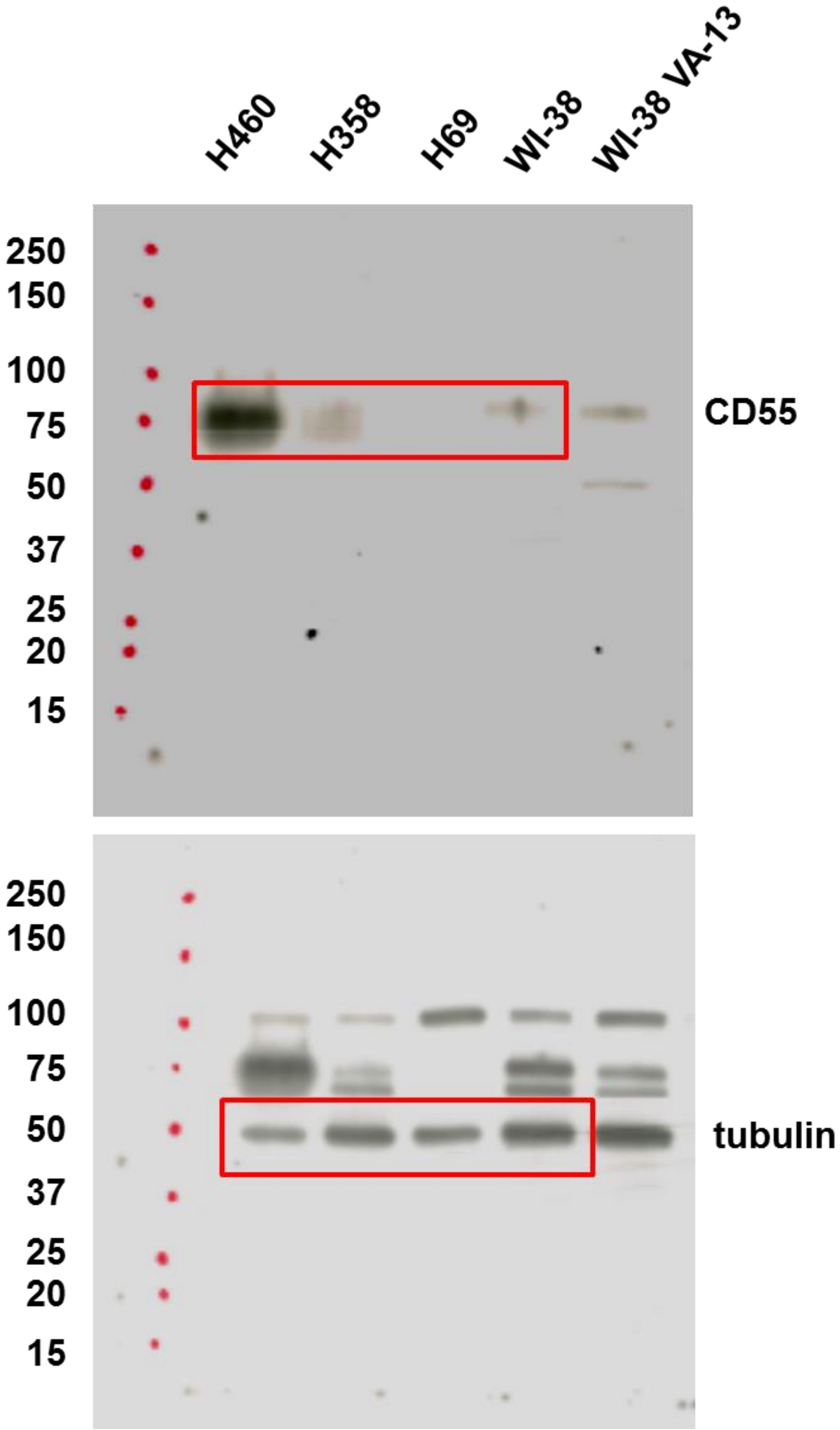

Supplementarty Figure S14. Full-length Western blots corresponding to the cropped images shown in Supplementary Fig. S2.

Supplementary Table S1. Expression of CD55 in NSCLC tissues. Immunohistochemical quantification of CD55 in NSCLC tissues. Strong, moderate, and negative indicate >50%, 10-50%, and <10%, respectively.

Clinical types of NSCLC	Number of stained tissues			Total positive tissues
	Strong	Moderate	Negative	
Squamous cell carcinoma	3/12	7/12	2/12	10/12
Adenocarcinoma	4/7	1/7	2/7	5/7
Bronchioloalveolar carcinoma	1/4	1/4	2/4	2/4
Adenosquamous carcinoma	2/3	1/3	0/3	3/3
Large cell carcinoma	2/3	1/3	0/3	3/3
Mucoepidermoid carcinoma	2/3	0/3	1/3	2/3
Mucinous adenocarcinoma	0/1	0/1	1/1	0/1
Adenoid cystic carcinoma	0/1	1/1	0/1	1/1

References

1 Lim, J. C. *et al.* Preclinical pharmacokinetic, biodistribution, imaging and therapeutic efficacy of (177)Lu-Labeled glycated bombesin analogue for gastrin-releasing peptide receptor-positive prostate tumor targeting. *Nucl Med Biol* **42**, 234-241, doi:10.1016/j.nucmedbio.2014.10.008 (2015).
